# *Inonotus obliquus* Polysaccharides: Preparation, Structural Characteristics, Structure–Activity Relationships, Biological Activities and Applications

**DOI:** 10.3390/nu18071125

**Published:** 2026-03-31

**Authors:** Siying Zhang, Wenshuo Zhang, Xue Wu, Shouchen Li, Dongyuan Shi, Hongyu Li, Ting Liu, Aihua Gong

**Affiliations:** 1 School of Medicine, Jiangsu University, Zhenjiang 212013, China; zhangsy359@ujs.edu.cn (S.Z.); 3221501026@stmail.ujs.edu.cn (W.Z.); 3221501012@stmail.ujs.edu.cn (X.W.); 3221501023@stmail.ujs.edu.cn (S.L.); 3221501025@stmail.ujs.edu.cn (D.S.); 3221501019@stmail.ujs.edu.cn (H.L.); 2 Luzhou Center for Disease Control and Prevention, Luzhou 646300, China; liut700@nenu.edu.cn

**Keywords:** *Inonotus obliquus* polysaccharides, isolation and purification, structure–activity relationships, biological activity

## Abstract

*Inonotus obliquus*, a rare medicinal and edible fungus, is rich in bioactive polysaccharides. These polysaccharides exhibit diverse biological activities, including gut microbiota-modulating, hypoglycemic, immunomodulatory, antitumor, antioxidant, hypolipidemic, and antiviral activities. Owing to these remarkable bioactivities and favorable safety profiles, *Inonotus obliquus* polysaccharides (IOPs) have garnered considerable research interest as promising candidates for the development of functional foods and therapeutic agents, particularly for the management of metabolic diseases and cancers. Based on the latest advances in the research on IOPs, this review summarizes its isolation and purification methods, structural characteristics, structure–activity relationships, biological properties and mechanisms, as well as their potential applications. It aims to provide valuable theoretical references for the further development and practical application of IOPs in the fields of medicine and functional foods.

## 1. Introduction

Mushrooms, as macrofungi with distinct fruiting bodies and mycelia, are renowned not only for their culinary value but also for their diverse health-promoting properties. Nutritionally, they are rich in essential components such as polysaccharides, polyphenols, proteins, niacin, potassium, riboflavin, selenium, vitamin D, and dietary fiber [1]. Beyond their nutritional value, numerous mushroom species have been extensively studied for their medicinal benefits, including anti-cancer, anti-inflammatory, and immunomodulatory effects, as well as protective actions against metabolic diseases and degenerative brain disorders [2]. *Inonotus obliquus* (Chaga mushroom) is an important edible mushroom that parasitizes the trunk of birch trees [3] and belongs to the family Hymenochaetacea of Basidiomycotina [4]. Morphologically, it is characterized by a dark, irregular mass with a charred texture [3]. Geographically, *Inonotus obliquus* is primarily distributed in cold regions of the Northern Hemisphere (40–68° N latitude), including North America, Finland, Poland, Russia, Japan, as well as Heilongjiang Province and Jilin Province of China [5,6]. Notably, its growth rate is extremely slow [7], making it a scarce and valuable wild mushroom resource. The main bioactive components of *Inonotus obliquus* are polysaccharides [7], triterpenoids [8,9], polyphenols [10], steroids [11], and melanins [12]. Since the 16th century, *Inonotus obliquus* has been used in Russia and other Eastern European countries to treat gastrointestinal cancer, cardiovascular disease, and diabetes, with no significant toxic side effects reported [6]. Furthermore, studies have confirmed its diverse biological activities, such as antitumor, antiviral, antioxidant, hypolipidemic, immunomodulatory, and anti-inflammatory activities [13]. Owing to its remarkable and diverse healthcare and biological effects, *Inonotus obliquus* has attracted considerable attention from researchers worldwide.

IOPs are regarded as the most bioactive ingredient among all bioactive substances of *Inonotus obliquus* [14], with their bioactivity strongly dependent on monosaccharide composition and structural characteristics [7]. As early as the 1960s, aqueous extracts of *Inonotus obliquus* were first reported to exhibit tumor-suppressive activity [15]. Since then, in-depth research has revealed that IOPs possess a broad spectrum of biological effects, including gut microbiota-modulating, hypoglycemic, immunomodulatory, antitumor, hypolipidemic, and antiviral activities [7]. Notably, the diverse biological activities of IOPs are closely associated with its extraction methods and structural characteristics.

Advances in analytical technologies have significantly expanded the research scope of IOPs. However, the number of relevant review articles remains limited, and existing ones often exhibit a narrow focus, outdated information, and insufficient systematic analysis of structure–activity relationships. In particular, recent reviews have primarily focused on the biological activities of IOPs while providing limited insights into the mechanisms and comprehensive structure–activity relationships. To address these gaps, this review consolidates current research findings to provide a thorough overview of the preparation, structural characteristics, structure–activity relationships, biological effects, and applications of IOPs. Furthermore, current research challenges and future prospects of IOPs are also discussed.

A systematic literature search was conducted in major international databases, including Web of Science, ScienceDirect, and PubMed, to identify studies on *Inonotus obliquus* and its bioactive polysaccharides. More than 84% of the references included in this review were published between January 2015 and November 2025. The search terms included “*Inonotus obliquus*”, “Chaga”, “*Inonotus obliquus* polysaccharides”, “*Inonotus obliquus* polysaccharides structure”, “*Inonotus obliquus* polysaccharides and gut microbiota modulation”, “*Inonotus obliquus* polysaccharides and hypoglycemic”, “*Inonotus obliquus* polysaccharides and hypolipidemic”, “*Inonotus obliquus* polysaccharides and immunomodulatory”, “*Inonotus obliquus* polysaccharides and antitumor”, “*Inonotus obliquus* polysaccharides and antioxidant”, and “*Inonotus obliquus* polysaccharides and antiviral”. The initial search yielded approximately 250 records. After removing duplicate records, the remaining articles were screened based on their titles and abstracts, resulting in the inclusion of approximately 100 relevant publications. Particular focus was placed on selecting studies that provided insights into the structural features of IOPs and their connection to biological functions, especially concerning gut microbiota modulation.

## 2. Preparation of IOPs

### 2.1. Extraction Method of IOPs

The structure and biological activity of polysaccharides are influenced by the extraction methods [16]. Currently, the primary extraction method for IOPs is hot water extraction. Other methods include subcritical water extraction as well as auxiliary extraction techniques such as ultrasonic-assisted extraction and microwave-assisted extraction. The characteristics of different extraction methods for IOPs are shown in Table 1. To overcome the limitations of single extraction methods, such as polysaccharide degradation and low efficiency, combined extraction technology has been developed. Through the complementary methods, the extraction efficiency and yield of IOPs can be synergistically improved while preserving their active structures. For instance, Che et al. [17] used ultrasound/microwave-assisted extraction to obtain IOPs, with the optimal conditions being a microwave power of 90 W, an ultrasonic power of 50 W, an ultrasonic frequency of 40 kHz, an extraction time of 19 min, and a solid-to-liquid ratio of 1:20 g·mL^−1^. This technique not only markedly reduced the extraction time over hot water extraction but also enhanced the polysaccharide yield (from 2% to 3%) and purity (from 64% to 73%).

**Table 1:** Characteristics of different extraction methods for IOPs.

Extraction Method	Principle	Advantages	Disadvantages	Conditions	Polysaccharide Yield	References
Hot water extraction	Water penetration into cells and dissolution of polysaccharides	Simple operation, low equipment cost, no pollution	Time-consuming, low efficiency, numerous water-soluble impurities	Temperature: 80 °C, Time: 2 h, Solid–liquid ratio: 1:20 g·mL^−1^	4%	[18]
				Temperature: 100 °C, Time: 4 h, Solid–liquid ratio: 1:10 g·mL^−1^	7%	[19]
Alkaline extraction	Degradation of cell wall components by hydroxide ions	High yield, high purity, broad applicability	Potential polysaccharide bioactivity loss, high reagent and equipment costs, complex processing steps	1 M NaOH, Temperature: 100 °C, Time: 4 h, Solid–liquid ratio: -	8%	[20]
Ultrasound-assisted extraction	Cell wall disruption via cavitation, enhancing solvent penetration	High efficiency, reduced extraction time, simple equipment	Potential polysaccharide degradation, structural alteration	Temperature: 63 °C, Ultrasonic frequency: 28 kHz, Time: 31 min, Solid–liquid ratio: 1:20 g·mL^−1^	4%	[21]
Microwave-assisted extraction	Rapid internal heating via electromagnetic energy, causing cell rupture	Rapid, efficient, low solvent consumption	Risk of structural changes, potential for uneven heating	Temperature: 61 °C, Time: 64 min, Solid–liquid ratio: 1:30 g·mL^−1^	22%	[22]
Subcritical water extraction	Reduced dielectric constant of water under high temperature and pressure, facilitating the dissolution of polysaccharides	High extraction and modification efficiency, no residue or wastewater, environmentally friendly	High requirements for temperature and pressure	Temperature: 200 °C, Time: 13.40 min, Solid–liquid ratio: 1:30 g·mL^−1^	14%	[18]
Ultrasound/microwave-assisted extraction	Synergistic combination of ultrasonic cavitation and microwave heating	Reduced extraction time, enhanced polysaccharide yield and purity	High equipment cost, degradation of the polysaccharide structure	Microwave power: 90 W, Ultrasonic power: 50 W, Ultrasonic frequency: 40 kHz, Time: 19 min, Solid–liquid ratio: 1:20 g·mL^−1^	3%	[17]

### 2.2. Purification Method of IOPs

#### 2.2.1. Enrichment of IOPs

Crude extracts of IOPs typically contain various impurities such as proteins and pigments, which can affect their biological activity. Thus, it is essential to remove these unwanted components to enrich crude IOPs. Enrichment methods for IOPs primarily include deproteinization and depigmentation. Common deproteinization methods for IOPs include the Sevage method, trichloroacetic acid (TCA) method, and enzymatic methods [23]. Common depigmentation methods include activated carbon adsorption, hydrogen peroxide (H_2_O_2_) oxidation, and ion-exchange resins [24]. The characteristics of these methods are listed in Table 2.

It is worth noting that there is a novel enrichment technique—three-phase partitioning (TPP) [25]. In this method, (NH_4_)_2_SO_4_ is mixed with the crude extract, followed by the addition of t-butanol, resulting in three distinct phases. The upper organic phase contains t-butanol, concentrated pigments, lipids, and hydrophobic materials; the middle phase consists primarily of proteins, while (NH_4_)_2_SO_4_ and IOPs are concentrated in the lower phase. Thus, TPP integrates deproteinization and depigmentation into a single step, offering an efficient, rapid, and environmentally friendly alternative to conventional multi-step purification procedures.

#### 2.2.2. Purification of IOP Fractions

Common methods for purifying IOPs include ethanol fractionation precipitation and column chromatography. In ethanol fractionation, polysaccharides can be sequentially precipitated by increasing the ethanol concentration. For instance, fractions designated as IOP40, IOP60, and IOP80 are obtained at 40%, 60%, and 80% ethanol concentrations, respectively [26]. Regarding column chromatography, both ion-exchange chromatography and gel filtration chromatography are widely applied, with their respective characteristics detailed in Table 2. Xu et al. [27] purified IOPs using a DEAE-52 cellulose column (50 cm × 2.6 cm), eluting with 0 M, 0.25 M, and 0.5 M NaCl solutions at a flow rate of 0.4 mL·min^−1^. This process yielded three polysaccharide fractions with different charge properties, designated IOP1, IOP2, and IOP3. Subsequently, IOP2 was further purified by Sephadex G-100 gel column chromatography (1.6 cm × 40 cm) eluted with 0.15 M NaCl at a flow rate of 0.18 mL·min^−1^. This step produced a homogeneous fraction named IOP2-A, which exhibited uniform charge and molecular weight (Mw) characteristics, with an Mw of 42 kDa.

## 3. Structural Characteristics of Polysaccharides

The biological activity of IOPs is closely related to their structure, making their structural analysis particularly important. Polysaccharide structures are categorized into primary and advanced structures. The primary structure comprises molecular weight, monosaccharide composition, glycosidic linkages, and anomeric configuration (α or β). The advanced structure of polysaccharides is primarily composed of triple-helical and crystalline conformations [16].

A suite of analytical techniques is employed to decipher this structural complexity. Currently, size exclusion chromatography (SEC) and gel permeation chromatography (GPC) are the predominant techniques for determining Mw [28]. The initial step in analyzing monosaccharide composition is to hydrolyze the polysaccharide via total acid hydrolysis [29]. After hydrolysis, high-performance liquid chromatography (HPLC) and gas chromatography (GC) represent the predominant techniques for monosaccharide analysis [30,31]. Elucidating the glycosidic linkages between monosaccharides requires more intricate procedures. Comprehensive pretreatment is required, including methylation, periodate oxidation, and Smith degradation, followed by gas chromatography-mass spectrometry (GC-MS) to determine the composition of glycosidic linkages [16]. Furthermore, nuclear magnetic resonance (NMR) spectroscopy is indispensable for determining the types of glycosidic bonds and sequence compositions. One-dimensional (1D) NMR, specifically ^1^H and ^13^C NMR spectroscopy, is routinely employed for the characterization of polysaccharides. A key application is the identification of the anomeric configuration (α- or β-) of sugar residues, which is discernible from the characteristic chemical shifts of the anomeric proton (H1) and the anomeric carbon (C1). For detailed structural elucidation, a suite of two-dimensional (2D) NMR techniques is essential. Key experiments include the following;: correlation spectroscopy (COSY) reveals through-bond scalar couplings between vicinal protons within a sugar residue, enabling the stepwise assignment of its proton spin system; total correlation spectroscopy (TOCSY) provides correlations among all protons belonging to the same spin system; and heteronuclear single quantum correlation spectroscopy (HSQC) or heteronuclear multiple quantum correlation spectroscopy (HMQC) spectra show the direct one-bond heteronuclear correlations (^1^JCH) between a proton and its directly attached carbon, which are fundamental for assigning the ^1^H and ^13^C chemical shifts of each monosaccharide unit. In practice, HSQC is more widely employed in modern polysaccharide research due to its superior resolution in crowded spectral regions, being less susceptible to signal broadening caused by proton–proton scalar couplings. Nuclear Overhauser effect spectroscopy (NOESY) and rotating-frame Overhauser effect spectroscopy (ROESY) detect through-space proximities between protons via the nuclear Overhauser effect (NOE), providing critical information on molecular conformation. For medium-sized polysaccharides, ROESY is generally the preferred and more reliable technique because it overcomes the “zero-crossing” signal limitation that can render conventional NOESY ineffective. Finally, heteronuclear multiple bond correlation spectroscopy (HMBC) reveals long-range heteronuclear couplings (typically over two or three bonds, ^2,3^JCH) and is indispensable for establishing the sequence of glycosidic linkages by correlating the anomeric proton of one residue with the aglycone carbon of an adjacent residue [32,33,34,35,36]. Moreover, IR spectroscopy can detect the functional groups present in polysaccharides [28]. The purity of polysaccharide can be assessed by ultraviolet-visible (UV–Vis) spectroscopy, which can determine whether proteins and nucleic acids are present [18,37]. Regarding advanced structures, the crystal structure is commonly analyzed by X-ray diffraction techniques [16]. The Congo red assay is employed to characterize the triple-helix conformation of polysaccharides, based on the principle that Congo red forms specific complexes with triple-helical polysaccharide structures. Upon increasing the concentration of sodium hydroxide (NaOH), these polysaccharide-Congo red complexes gradually dissociate, which is accompanied by a characteristic shift in the maximum absorption wavelength (λmax). By monitoring the dynamic change of λmax, the integrity of the triple-helical structure can be evaluated. The resulting λmax versus NaOH concentration plot typically reveals four distinct zones corresponding to undefined region, stable triple-helix, conformational transition, and random coil states. This method provides valuable insights into polysaccharide higher-order structure, though it should be noted that the analytical accuracy of the Congo red assay remains a subject of debate, which necessitates careful interpretation of the obtained results [38,39].

A fundamental and persistent challenge in the study of IOPs is the difficulty of achieving complete and unambiguous structural elucidation, even for purified fractions, despite the application of advanced complementary analytical techniques. This is clearly evidenced by the fact that, to date, no study has presented a fully defined chemical structure for an isolated, homogeneous IOP fraction. Consequently, the structural models reported in the literature, including those summarized in this review, should be regarded as the most plausible interpretations derived from the integration of complementary analytical data (e.g., monosaccharide composition, glycosidic linkage analysis via methylation/GC-MS, and NMR spectral assignments). Therefore, the structural characterizations discussed herein predominantly reflect the collective or average features of IOP mixtures, rather than the definitive architecture of a single molecular entity. This inherent methodological limitation, which underscores the complexity of native polysaccharides, must be carefully considered when interpreting structural data and proposing structure–activity relationships.

**Figure 1:**
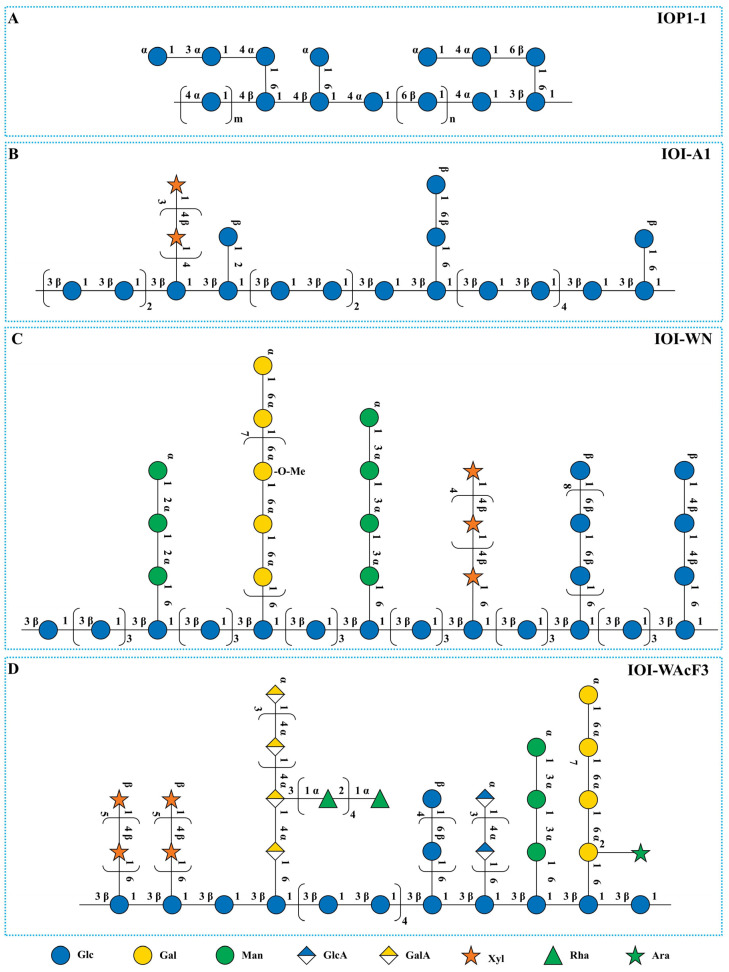
Representative structural models of neutral (**A**–**C**) and acidic (**D**) IOPs [20,37]. These schematic models were derived from GC, GC-MS and NMR analyses. They illustrate the main structural features but do not represent the actual three-dimensional conformation or precise glycosidic linkages of the polysaccharides. Some minor sugar residues accounting for less than 2% in methylation analysis are not depicted.

Four possible structures proposed for IOPs are presented in Figure 1. Ding et al. [37] reported a neutral polysaccharide, IOP1-1 (Figure 1A), which was isolated via hot water extraction and purified by DEAE-52 cellulose and Sephadex G-100 chromatography. Structural analysis revealed that IOP1-1 had an Mw of 6886 Da and was composed of D-glucose. A comparison of IOP1-1 with other reported polysaccharides in terms of molecular weight, monosaccharide composition, and structural features suggests that it may represent a novel polysaccharide isolated from the black crystal region of *Inonotus obliquus*. The black crystal part was obtained from the surface layer of *Inonotus obliquus* fruiting body. Fourier transform infrared (FTIR) spectroscopy indicated that IOP1-1 was a pyranose-type polysaccharide potentially containing both α- and β-glycosidic bonds. Methylation analysis revealed that IOP1-1 contained 1,4-, 1-, 1,6-, 1,4,6-, 1,3-, 1,3,6-, 1,2-, 1,2,6-, 1,2,4-, 1,3,4-, 1,2,3-, and 1,3,4,6-linked glucose (Glc). NMR spectroscopy confirmed α- and β-anomeric configurations and identified a main chain structure of α-Glc*p*-(1 → 4)-α-Glc*p*-(1 → 4)-β-Glc*p*-(1 → 4)-β-Glc*p*-(1 → 4)-α-Glc*p*-(1 → 6)-β-Glc*p*-(1 → 4)-α-Glc*p*-(1 → 3)-β-Glc*p*-(1 → (Figure 1A).

In contrast, neutral IOI-A1 (Figure 1B) was isolated through alkaline extraction followed by purification via Sephacryl S-500 h column chromatography. IOI-A1 has an average molecular weight exceeding 450 kDa. Monosaccharide composition analysis revealed that IOI-A1 is primarily composed of glucose (79.2%) and xylose (12.1%), accompanied by trace amounts of mannose, fucose, and N-acetylglucosamine. The GC-MS analysis indicated that IOI-A1 mainly contains (1 → 3)-linked glucose (57.3%), along with minor proportions of (1 → 6)-linked glucose (3.3%), (1 → 3,6)-linked glucose (5.6%), and terminal glucose (8%). Combined with nuclear magnetic resonance (NMR) spectral data, these results suggest that IOI-A1 is a polymeric structure with a backbone consisting of (1 → 3)-linked β-glucose residues, which are decorated with short side chains of (1 → 6)-linked β-glucose; additionally, β-xylose residues may be substituted between these sugar moieties [20].

Another neutral polysaccharide, designated IOI-WN (Figure 1C), was isolated through hot water extraction followed by purification via ANX Sepharose anion-exchange chromatography. This polymer has an average molecular weight of 60 kDa. Monosaccharide composition analysis indicated that IOI-WN was mainly composed of glucose (53.1%), galactose (17.5%), xylose (6.9%), mannose (6.8%), 3-O-methylated galactose (8.7%), together with small amounts of arabinose, rhamnose, fucose, glucuronic acid, and galacturonic acid. Methylation/GC-MS analysis indicated a heterogeneous, highly branched configuration. Specifically, glucose was mainly (1 → 3) and (1 → 6) linked, galactose was almost entirely (1 → 6) linked, and mannose was mainly (1 → 2) and (1 → 3) linked. Additionally, minor components were identified, including (1 → 4)-linked xylose, (1 → 2)-linked mannose, (1 → 3)-linked mannose, terminal arabinose, and terminal fucose. Furthermore, NMR spectroscopy elucidated a backbone of (1 → 3)-linked β-Glc residues with (1 → 6)-linked β-Glc kinks at approximately every fifth residue intervals, and branches including (1 → 6)-linked β-Glc and (1 → 6)-linked 3-O-methylated α-galactose per approximately every third galactose residue (Figure 1C) [20]. It remains to be further clarified whether these minor, diverse monosaccharides are covalently linked to the galactoglucan backbone or branches alongside glucose and galactose, or whether they exist as independent polysaccharide chains co-existing with the major galactoglucan that cannot be separated by anion-exchange and size-exclusion chromatography. In the latter case, partial acid hydrolysis, enzymatic hydrolysis, or other advanced structural elucidation methods will be essential to distinguish and characterize these potential minor polysaccharide components.

The acidic polysaccharide IOI-WAcF3 (Figure 1D), with an average Mw of 10 kDa, was isolated via hot water extraction, ANX Sepharose anion-exchange, and Superdex 200 size exclusion chromatography. Monosaccharide composition analysis indicated that IOI-WAcF3 was mainly composed of glucose (39.2%), xylose (19.0%), galacturonic acid (12.9%), galactose (8.1%), rhamnose (7.7%), mannose (4.8%), and glucuronic acid (4.4%), together with minor amounts of arabinose, fucose, and 3-O-methylated galactose. Methylation/GC-MS analysis demonstrated a heterogeneous, branched structure: glucose was mainly (1 → 3) and (1 → 6) linked, galacturonic acid was predominantly (1 → 4)-linked, and glucuronic acid was almost entirely (1 → 4) linked. Additionally, minor constituents included (1 → 3)-linked mannose, (1 → 6)-linked galactose, (1 → 4)-linked xylose, and terminal arabinose. NMR spectroscopy indicated a backbone of (1 → 3)-linked β-Glc residues with (1 → 6)-linked β-Glc kinks at approximately every fifth residue intervals, and branches including (1 → 6)-linked β-Glc, (1 → 4)-linked α-galacturonic acid and (1 → 4)-linked α-glucuronic acid (Figure 1D) [20]. While the GC-MS data suggest that the branching points in these polymers are primarily located on glucose residues—implying that these residues may serve as crucial junctions connecting different polymer segments—this specific linkage detail could not be directly confirmed by NMR spectroscopy. A critical point arises when comparing its structure with the provided bioactivity data. While IOI-WAcF3 (Figure 1D) shares the core (1 → 3)-β-Glc backbone with the highly active neutral fraction IOE-WN (Figure 1C), its immunomodulatory effect was less potent. This suggests that uronic acids confer acidic character but do not necessarily enhance, and may even modulate, this immune pathway. The negative charges from glucuronic acid or galacturonic acid could alter the three-dimensional conformation or electrostatic interactions with pattern recognition receptors, potentially explaining the different bioactivity profile compared to the neutral glucan. This observation underscores that fine structural details beyond monosaccharide composition are crucial for activity.

In contrast, another acidic polysaccharide IOP-2, with an average Mw of 26.13 kDa, was obtained by microwave-assisted extraction, ethanol precipitation, a DEAE-50 cellulose anion exchange column, and a Sephadex G-100 size exclusion chromatography column. Monosaccharide composition analysis via 1-phenyl-3-methyl-5-pyrazolone (PMP)-HPLC indicated that IOP-2 was composed of glucose (31.65%), galactose (18.72%), fucose (9.33%), arabinose (9.27%), mannose (10.70%), glucuronic acid (8.58%), galacturonic acid (4.98%), rhamnose (4.24%), and a minor amount of xylose. Methylation and GC-MS analysis further identified 17 distinct glycosidic linkages, with the major bonds being 1,3-linked Glc*p* (17.36%), 1,4-linked Glc*p* (14.85%), 1,4,6-linked Glc*p* (11.77%), and 1,4-linked Gal*p* (11.77%) [22]. As both IOP-2 and IOI-WAcF3 are acidic polysaccharides from *Inonotus obliquus*, what might account for their distinct structures? IOP-2 was obtained using a holistic strategy. The starting material was undifferentiated, whole sclerotium powder, meaning that the entire Chaga sclerotium was uniformly ground. Consequently, the IOP-2 polysaccharide represents an acidic fraction obtainable from the whole, mixed sclerotium. In contrast, the isolation of IOI-WAcF3 relied on anatomical dissection. Prior to extraction, the sclerotium was separated into a “brown interior” and a “black exterior”. IOI-WAcF3 was subsequently extracted exclusively from the interior tissue. Thus, IOI-WAcF3 is defined as an acidic polysaccharide subfraction specific to the inner tissue. Notably, several studies have also indicated that structural variations among polysaccharides are often attributed to differences in isolation methodologies, the geographic origin and age of *Inonotus obliquus*, or the specific tissue site selected for extraction.

Studies have demonstrated a positive correlation between the integrity of the triple-helical conformation of polysaccharides and their biological activity [40,41]. The crystal structure is related to the physicochemical properties of polysaccharides, such as viscosity, solubility, swelling, and flexibility [42]. According to Xie et al. [22], IOPs were identified as having a triple-helix structure via Congo red staining, along with semi-crystalline characteristics revealed by X-ray diffraction. Yan et al. [43] reported that the homogeneous polysaccharide DPG-2, isolated from *Inonotus obliquus*, had an Mw of 17.02 kDa and was primarily composed of glucose. The glucose units were identified as pyranose sugars connected via β-glycosidic bonds, specifically involving (1 → 6) and (1 → 3) linkages. Furthermore, DPG-2 was found to exhibit an ordered triple-helix conformation in neutral to weakly alkaline conditions. This specific conformation not only enhances polysaccharide stability but also prolongs their functional duration in vivo, such as in inhibiting α-glucosidase. Notably, the triple-helix conformation of polysaccharides is sensitive to drying methods. For instance, in a study on IOPs, samples prepared by hot air drying and freeze-drying exhibited a triple-helix structure under low alkali concentrations, whereas those subjected to vacuum drying failed to form such a structure [44].

## 4. Structure–Activity Relationships

The biological activity of IOPs is directly related to its physicochemical properties, including Mw, glycosidic linkages, monosaccharide composition, chemical modification, and polysaccharide–protein complexes. The structure–activity relationships of IOPs are summarized in Table 3. Understanding these relationships is crucial for establishing a foundation for the rational design of targeted therapeutics and functional foods.

### 4.1. Molecular Weight

Mw is one of the most established chemical characteristics impacting biological activity [45]. Generally, polysaccharides with lower molecular weights tend to exhibit stronger biological activities, while those with high molecular weights possess properties such as high viscosity and low solubility that can limit diffusion into biological tissues [46]. This relationship was illustrated in a study investigating the effects of in vitro digestion on the biological activities of polysaccharides from *Inonotus obliquus* (UIOPS-1) [47]. UIOPS-1 had an initial Mw of 105.02 kDa and was composed of 77.99% neutral sugar, 10% uronic acid, and 9.32% protein. Its monosaccharide composition consisted of L-rhamnose, D-arabinose, D-xylose, D-mannose, D-glucose, and D-galactose in molar ratios of 1.49, 2.18, 1.96, 2.08, 1.94, and 2.46, respectively. Morphologically, UIOPS-1 exhibited a polygonal and flake-like shape with neat edges and a smooth surface. Following gastric digestion, the Mw of the resulting fraction (UIOPS-1G) decreased to 97.08 kDa. UIOPS-1G contained 79.09% neutral sugar, 9.62% uronic acid, and 8.48% protein. Its monosaccharide composition consisted of L-rhamnose, D-arabinose, D-xylose, D-mannose, D-glucose, and D-galactose in molar ratios of 1.08, 1.46, 1.15, 0.98, 1.09, and 1.13, respectively. Morphologically, UIOPS-1G exhibited a rough surface with distinct furrows. Compared to UIOPS-1, UIOPS-1G exhibited significantly increased antioxidant activity, along with enhanced inhibitory activities against α-amylase and α-glucosidase. After intestinal digestion, the resulting fraction (UIOPS-1I) underwent further Mw reduction, yielding two sub-fractions with MW values of 88.97 kDa and 36.74 kDa. UIOPS-1I contained 72.38% neutral sugar, 8.04% uronic acid, and 8.19% protein. Its monosaccharide composition consisted of L-rhamnose, D-arabinose, D-xylose, D-mannose, D-glucose, and D-galactose in molar ratios of 2.95, 3.80, 2.42, 1.87, 2.10, and 2.30, respectively. Morphologically, UIOPS-1I appeared as small pieces with particles on the surface and exhibited a porous honeycomb structure. In contrast to UIOPS-1, UIOPS-1I showed reduced antioxidant activity but significantly increased the inhibitory activities against both α-amylase and α-glucosidase. Thus, the Mw range required to achieve maximum bioactivity varies according to the specific biological function. However, in another study evaluating the antioxidant activities of polysaccharides from *Inonotus obliquus*, IOP2a had an average Mw of 93 kDa and contained 91.4% total sugar and 7.5% uronic acid. The monosaccharide composition of IOP2a consisted of rhamnose, mannose, and glucose, with molar ratios of 1.0:2.3:1.7, respectively. In contrast, IOP2c exhibited an average Mw of 230 kDa and contained 96.5% total sugar and 7.7% uronic acid, with its monosaccharide composition comprising mannose, glucose, and galactose at a molar ratio of 1.0:2.9:0.8. Although IOP2a and IOP2c had similar uronic acid contents, IOP2c, with a relatively higher Mw, demonstrated significantly greater hydroxyl radical scavenging activity than IOP2a [48].

### 4.2. Glycosidic Linkages

Beyond Mw, the presence of (1 → 3)/(1 → 6)-β-Glc motifs within the IOPs is important for bioactivity. The neutral, acidic, and alkaline polysaccharide fractions isolated from *Inonotus obliquus*, all of which contained (1 → 3) and (1 → 6)-β-Glc motifs, showed in vitro immunomodulatory effect by increasing nitric oxide (NO) production in the murine macrophage and dendritic cell lines J774.A1 and D2SC/1 [20]. Similarly, Araújo-Rodrigues et al. [45] concluded that mushroom β-glucans with linear β-(1 → 3) and β-(1 → 6) linkages may possess strong immunomodulatory potential. Furthermore, the *Inonotus obliquus* polysaccharide DPG-2 demonstrated an enhanced α-glucosidase inhibitory activity, which was attributed to its higher proportion of β-(1 → 3) and β-(1 → 6) glycosidic bonds [43].

### 4.3. Monosaccharide Composition

The antioxidant activity of polysaccharides is closely associated with their monosaccharide composition, including the content of arabinose and uronic acid. Pearson correlation analysis revealed a relationship between the arabinose content in IOPs and 1,1-diphenyl-2-picrylhydrazyl (DPPH) scavenging activity: specifically, IOP fractions with lower arabinose content exhibited stronger antioxidant capacity [49]. This observation contrasts with findings on lotus root polysaccharides, where arabinose exerted a positive effect on DPPH radical scavenging [50]. However, the antioxidant activity attributable to free reducing sugar end groups is inherently weak. In contrast, arabinose–phenolic acid complexes may exert significant antioxidant effects through three distinct mechanisms: (i) phenolic acid-mediated free radical scavenging via hydrogen atom transfer from catechol/phenol groups; (ii) inhibition of lipid peroxidation through interruption of radical chain reactions by phenolic moieties; and (iii) metal ion chelation involving the coordination of transition metals by phenolic hydroxyls and carboxyls to prevent Fenton reactions. Consequently, the observed antioxidant activity of this fraction is presumably attributable to its bound phenolic acids [51]. These conflicting results indicate that arabinose content exerts a dual, context-dependent influence on polysaccharide antioxidant activity. In contrast to arabinose, uronic acid content consistently shows a positive correlation with the antioxidant potential of IOPs. Huang et al. [48] demonstrated that IOP3a and IOP4 rich in uronic acids exhibited potent hydroxyl radical scavenging activity, while IOP1b with no uronic acids exhibited lower scavenging activity.

### 4.4. Chemical Modification

Besides monosaccharide composition, chemical modification is a key strategy to enhance the antioxidant activity of IOPs. This aligns with broader findings in mushroom polysaccharide research, where modifications such as acetylation, carboxymethylation, and selenylation are widely reported to improve the bioactive properties [45]. According to Ma et al. [52], an acetylated *Inonotus obliquus* polysaccharide (Ac-IOPS), isolated via hot water extraction, could be explored as a novel antioxidant for human consumption. Ac-IOPS had an Mw of 103.2 kDa and contained 41.29% neutral sugar, 14.11% uronic acid, and 6.80% protein. It was mainly composed of glucose (73.20%), mannose (16.60%), and xylose (4.95%), together with minor monosaccharides including galactose, arabinose, and rhamnose at contents below 2%. Morphologically, Ac-IOPS exhibited a rough, island-like surface with numerous pores, suggesting a highly branched structure. Furthermore, Ac-IOPS exhibited significantly stronger ferric reducing power and greater inhibition of liver lipid peroxidation compared to the unmodified, sulfated, and carboxymethylated polysaccharide fractions. This was consistent with the results of Thimmaraju et al. [53], who found that an acetylated *Hypsizygus ulmarius* polysaccharide (Ac-HUP1) exhibited significantly enhanced antioxidant activity compared to its native counterpart (HUP1). However, Xie et al. [54] demonstrated that the carboxymethylated *Inonotus obliquus* polysaccharide (IOP-C), prepared via microwave-assisted extraction, showed promise as an edible antioxidant. IOP-C had an Mw of 244 kDa and contained 30.38% neutral sugar, 15.44% uronic acid, and 1.64% protein. It was mainly composed of glucose (35.46%), mannose (24.58%), glucuronic acid (9.23%), arabinose (6.78%), galactose (6.45%), rhamnose (5.62%), xylose (3.80%), and galacturonic acid (3.73%), with trace amounts of other minor saccharide components not fully identified in the original literature. Structurally, IOP-C exhibited a hybrid morphology comprising both crystalline and amorphous components, and it did not possess a triple-helical structure. Microscopically, IOP-C appeared as curled, flaky fragments with a rough surface dotted with small particles. Furthermore, IOP-C exhibited a stronger scavenging rate against ABTS radicals compared to the acetylated polysaccharide. The conflicting findings regarding whether acetylation or carboxymethylation confers superior antioxidant activity may be attributed to the distinct initial structural properties of the polysaccharides resulting from the different extraction methods. Selenylation also significantly enhances the antioxidant activity of IOPs. A comparative investigation revealed that a selenized *Inonotus obliquus* polysaccharide (Se-IOP) outperformed its native form (IOP) [55]. Se-IOP had an Mw of 28.071 kDa and was composed of mannose, glucose, and galactose in molar ratios of 8.3, 32.1, and 22.7, respectively. In vitro, Se-IOP demonstrated a superior capacity to scavenge hydroxyl, DPPH, and superoxide radicals, while in vivo it reduced malondialdehyde (MDA) levels and increased the activities of antioxidant enzymes, including superoxide dismutase (SOD) and glutathione peroxidase (GSH-Px). Collectively, acetylation, carboxymethylation, and selenylation can all enhance the antioxidant activity of IOPs, yet variations exist in their efficacy and functional advantages. Such differences may arise from how each modification alters the polysaccharide’s molecular structure and reactive properties, with these effects further influenced by the inherent structural characteristics of IOPs, which are shaped by the preparation methods.

### 4.5. Polysaccharide–Protein Complex

Polysaccharide–protein complex is also an important factor modulating antioxidant activity. Among the five crude IOPs fractions, IOP3a, with the highest content of proteinaceous material, exhibited the strongest superoxide radical scavenging activity [48]. This fraction IOP3a had an average Mw of 44 kDa and contained 83.6% of total sugar, 21.2% uronic acid, and 21.4% protein. The monosaccharide composition of IOP3a consisted of rhamnose, mannose, glucose and galactose, with molar ratios of 0.3:4.6:2.3:1.0, respectively. However, the interactions between the mushroom polysaccharides and proteins are not yet fully understood, and further targeted research is needed to elucidate their underlying mechanisms [56].

### 4.6. Lignin–Carbohydrate Complexes

When discussing the bioactive components of *Inonotus obliquus*, it is crucial to consider that its polysaccharides do not exist in isolation. A key aspect of their natural state is their tendency to form associations with other cell wall constituents, notably lignin. This leads to the formation of lignin–carbohydrate complexes (LCCs), which may possess distinct and potent bioactivities compared to their isolated components. Niu et al. [6] isolated and characterized three homogeneous LCCs (designated IOA1, IOA2, and IOA3) from an alkali extract of *Inonotus obliquus*. These complexes, with molecular weights of 6.1 × 10^4^, 2.9 × 10^4^, and 3.5 × 10^4^ g/mol, were primarily composed of lignin alongside approximately 25% carbohydrates. Monosaccharide composition analysis revealed that IOA1 was primarily composed of glucose, galactose, mannose, and xylose in a molar ratio of 9:1:1:4. IOA2 consisted mainly of glucose, galactose, and xylose in a molar ratio of 12:3:4, whereas IOA3 contained these three monosaccharides in a molar ratio of 5:1:2. Critically, this study demonstrated that these native complexes themselves exhibit pronounced in vitro antioxidant activity and significant immunostimulatory effects. This finding is pivotal, as it confirms that LCCs are not mere artifacts but are intrinsic, bioactive constituents of *Inonotus obliquus* with potential as natural antioxidants or immunostimulants. Therefore, the biological activities observed in many crude or semi-purified *Inonotus obliquus* extracts may be largely attributable to the synergistic action within these native complexes rather than to purified polysaccharides alone. Future research should prioritize the study of these composite structures, focusing on elucidating the structure–activity relationships specific to LCCs to fully understand the mechanistic basis of *Inonotus obliquus* efficacy and to advance the development of standardized extracts.

### 4.7. Other

In addition, the biological source of IOPs significantly influences the antitumor efficacy of the polysaccharides. For example, Jiang et al. [57] reported that a polysaccharide IOP extracted from wild *Inonotus obliquus* sclerotia exhibited in vitro antitumor activity. IOP had an average Mw of 45 kDa and was mainly composed of glucose (74.95%) and galacturonic acid (3.08%), together with small amounts of rhamnose, arabinose, and xylose. In contrast, the endo-polysaccharide FII-1, isolated from cultured *Inonotus obliquus* mycelium by Kim et al. [58], showed no in vitro antitumor activity. FII-1 had an average Mw of 1000 kDa and consisted of mannose, glucose, fucose, and glucosamine in molar ratios of 70.8:1.6:0.8:0.1. Structural analysis indicated that FII-1 contained α-glycosidic linkages. However, it should be noted that the observed differences in antitumor activity cannot be solely attributed to a single structural feature. The variations in biological source (wild sclerotia vs. cultured mycelium), molecular weight, monosaccharide composition, and glycosidic linkage patterns may contribute synergistically to the disparate bioactivities. Therefore, further systematic studies with standardized extraction and culture conditions are still required to clarify the precise structure–activity relationship of *Inonotus obliquus* polysaccharides.

**Table 3:** Relationships between the structure and activity of IOPs.

Fraction(s)	Mw (kDa)	Monosaccharide Composition and Molar Ratios (Mass Percentage or Molar Ratio)	Structural Features	Activity	Effects/Mechanisms	References
IOP-2S	26.76	Man:Rha:Glc:Gal:Xyl:Ara = 0.521:0.511:1.551:0.793:0.567:0.232	1,3-; 1,6-; 1,2- or 1,4- glycosidic bonds	Hypoglycemic	Inhibition of α-amylase and α-glucosidase	[26]
DPG-2	17.02	Glc	1,3-β-Glc*p*, 1,6-β-Glc*p*	Hypoglycemic	α-Glucosidase inhibition	[43]
HIOP1-S	13.6	Man:Rha:Glc:Gal:Xyl:Ara:Fuc = 1.75:12.232:29.673:20.547:2.386:15.786:17.626	α- and β-glycosidic bonds	Hypoglycemic	α-Glucosidase inhibition	[59]
HIOP2-S	15.2	Man:Rha:Glc:Gal:Xyl:Ara = 9.714:15.331:49.881:15.321:4.675:5.078	β-glycosidic bonds	Hypoglycemic	α-Glucosidase inhibition	[59]
IOE-WN	73	Ara:Rha:Fuc:Xyl:Man:Glc:Gal:GlcA:GalA:3-O-Me-Gal = 2.0:1.3:2.5:8.9:7.2:17.2:49.3:2.1:1.2:8.3	1,3/1,6-β-Glc*p*, 1,6-α-Gal*p*	Immunomodulatory	↑ NO production in J774.A1 and D2SC/1 cells	[20]
AcF1	28	Ara:Rha:Fuc:Xyl:Man:Glc:Gal:GlcA:GalA:3-O-Me-Gal = 2.3:4.8:1.5:4.7:10.8:13.5:34.3:4.4:17.7:6.0	1,3/1,6-β-Glc*p*, 1,6-α-Gal*p*, 1,4-α-GalA*p*	Immunomodulatory; antitumor	TLR2/4 activation; ↑ NO, TNF-α, IL-6	[60]
AcF3	10	Ara:Rha:Fuc:Xyl:Man:Glc:Gal:GlcA:GalA:3-O-Me-Gal = 2.3:7.7:0.6:19.0:4.8:8.1:39.2:4.3:12.9:1.1	1,3/1,6-β-Glc*p*, 1,6-α-Gal*p*, 1,4-α-GalA*p*, 1,4-β-Xyl*p*	Immunomodulatory; antitumor	TLR2/4 activation; ↑ NO, TNF-α, IL-6	[60]
IOP1-1	6.886	Glc	1,4-Glc*p*	Antitumor	Apoptosis via mitochondrial, death receptor, and ER stress pathways	[37]
IOP-1	12.67	Man:Rha:Glc:Gal:Xyl:Ara:Fuc = 26.06:2.35:46.32:11.44:4.78:7.07:1.98	1,4-Glc*p*, 1,3,6-Glc*p*, 1,3-Man*p*	Antioxidant	DPPH, ABTS,·OH scavenging; ferric ion reduction	[22]
IOP-2	26.13	Man:Rha:GlcA:GalA:Glc:Gal:Xyl:Ara:Fuc = 10.70:4.24:8.58:4.98:31.65:18.72:2.53:9.27:9.33	1,3-Glc*p*, 1,4-Glc*p*, 1,4,6-Glc*p*, 1,4-Gal*p*	Antioxidant	DPPH, ABTS,·OH scavenging; ferric ion reduction	[22]
IOP	32.5	Man:Rha:Glc:Gal:Xyl:Ara = 9.81:3.6:29.1:20.5:21.6:5.4	β-glycosidic bonds	Antioxidant	↑ SOD; ↓ MDA	[61]
IOP	42.28	Glc:Rha:Rib:GlcA:GalA = 85.2:3.91:1.73:2.14:1	β-Glc*p*	Hypolipidemic	AMPK activation; ↓ SREBP-1C, FAS, ACC; ↓ TC, TG, LDL-C	[62]
IOP-2A	1	Glc:Xyl:Gal:Man = 54.1:13.6:13.2:6.7	1,4-β-glycosidic bonds, 1,6-β-glycosidic bonds	Hypolipidemic; Gut microbiota modulation	↑ F/B ratio; ↓ fatty acid absorption	[63]
IOP	256.623	Man:Rib:Rha:GlcA:GalA:Glc:Gal:Xyl:Ara:Fuc = 0.011:0.005:0.006:0.016:0.002:1.000:0.008:0.003:0.004:0.001	1,4-α-Glc*p*	Androgenic; Gut microbiota modulation; Metabolic regulation	Lipid metabolism modulation; ↑ beneficial bacteria; ↓ obesity/diabetes-related metabolites	[64]

Abbreviations: Man, mannose; Rib, ribose; Rha, rhamnose; GlcA, glucuronic acid; GalA, galacturonic acid; Glc, glucose; Gal, galactose; Xyl, xylose; Ara, arabinose; Fuc, fucose; 3-O-Me-Gal, 3-O-methylated Gal; ↑, upregulate; ↓, downregulate.

## 5. Biological Activity of IOPs

**Figure 2:**
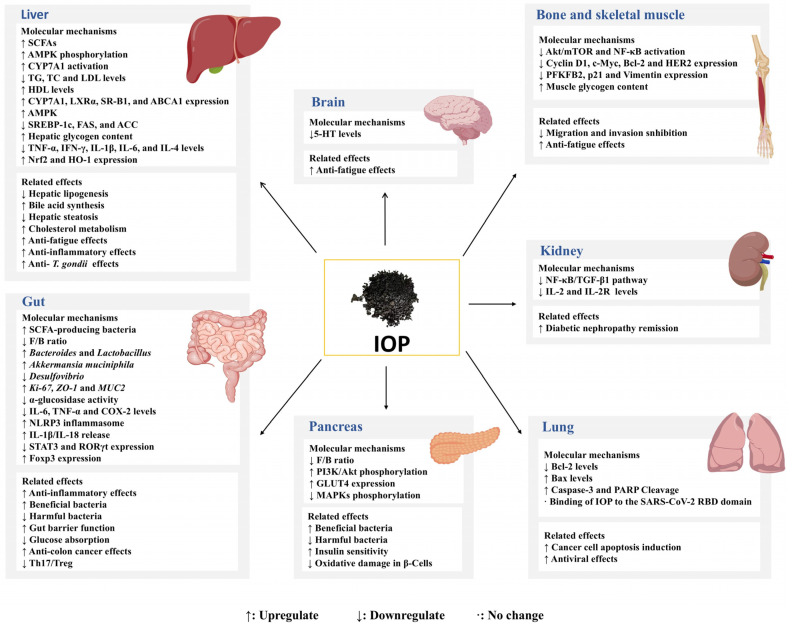
Molecular mechanisms and related biological effects of IOPs in multiple organs.

Recent studies on the bioactivities of IOPs have increasingly focused on their roles in modulating gut microbiota, as well as their hypoglycemic, immunomodulatory, and antitumor properties, as illustrated in Figure 2.

### 5.1. Gut Microbiota Modulation Activity

The gut microbiota plays a crucial role in maintaining intestinal homeostasis. Dysbiosis, an imbalance in this microbial community, is associated with intestinal, cardiovascular, metabolic, and neurological disorders [45]. IOPs demonstrate significant prebiotic potential by modulating the composition of the gut microbiota, thereby preserving intestinal homeostasis and strengthening mucosal immunity.

IOPs have been demonstrated to modulate gut microbiota composition in multiple animal models. Specifically, in the gut, as illustrated in the Figure 2, Hu et al. [65] demonstrated that IOP treatment reduced gut microbiota diversity and richness while decreasing *Firmicutes* and increasing *Bacteroidetes* abundance at the phylum level, shifting the microbial community toward a healthier profile in a chronic pancreatitis mouse model. Consequently, the Firmicutes/Bacteroidetes (F/B) ratio was significantly reduced. As depicted in Figure 2, Zhang et al. [66] similarly found that IOPs decreased the F/B ratio while increasing beneficial genera, including *Bacteroides* and *Lactobacillus*, in ulcerative colitis mice. As shown in Figure 2, Yang et al. [29] further demonstrated that IOPs selectively increased *Akkermansia muciniphila* while decreasing *Desulfovibrio*, indicating the targeted modulation of beneficial versus harmful bacteria.

Notably, IOPs exert sex-dependent effects on gut microbiota modulation. IOPs had different effects on the gut microbiota of male and female rats, with diversity and richness showing opposite changes. Following IOP intervention, alterations in dominant gut microbiota were less pronounced in female rats compared to males. Specifically, the F/B ratio decreased significantly in males but remained stable in females [67]. Liu et al. [68] further revealed that although inherent differences exist in cecal microbiota diversity between female and male rats, these differences were eliminated following IOP intake. They proposed that this IOP-mediated modulation of sex-dependent microbiota may be attributed to pH-dependent conformational changes in the structure of IOPs, influenced by sex hormones. Collectively, IOPs direct gut microbiota toward a health-promoting profile through the selective enrichment of beneficial bacteria and reduction in pathogenic populations.

### 5.2. Hypoglycemic Activity

According to the Global Burden of Disease Study, diabetes ranks as the ninth leading cause of reduced life expectancy [69]. Persistent hyperglycemia in type 2 diabetes mellitus (T2DM) damages multiple organs, giving rise to severe complications including cardiovascular disease, renal failure, and diabetic retinopathy [70]. Research indicates that IOPs possess excellent hypoglycemic property and may serve as a therapeutic agent for diabetes through multiple interconnected pathways, including microbiota-derived metabolite-mediated mechanisms.

In the gut, as illustrated in Figure 2, IOPs ameliorate metabolic diseases by enhancing intestinal barrier function. Specifically, IOPs repaired the intestinal barrier by upregulating the expression of *Ki-67, ZO-1*, and *MUC2* genes, thereby ameliorating diabetes mellitus [71]. In addition to microbiota-dependent mechanisms, IOPs lowered blood glucose through direct cellular and molecular pathways. In animal studies, IOP intervention ameliorated hyperglycemia and dyslipidemia in type 2 diabetic mice, accompanied by improved glucose tolerance and insulin sensitivity [72]. Further research demonstrated that the antihyperglycemic mechanism of IOPs involved activating PI3K and Akt phosphorylation as well as the translocation of glucose transporter 4 (GLUT4), as evidenced by the upregulated expression of PI3K-p85, phosphorylated Akt (Ser473), and GLUT4 in diabetic models, as shown in Figure 2 [73]. At the molecular level, in hepatic cells (Figure 2), two purified polysaccharide fractions from IOPs, HIOP1-S and HIOP2-S, enhanced glucose consumption in HepG2 cells and suppressed α-glucosidase activity, indicating a direct role in glycemic regulation [59].

Finally, IOPs alleviate diabetes-related complications. In renal tissue, as illustrated in Figure 2, low-molecular-weight IOPs slowed the progression of diabetic nephropathy in mice, potentially through anti-inflammatory and anti-fibrotic mechanisms involving the suppression of nuclear factor-κB (NF-κB) and TGF-β1 pathways [74]. Consistent with this, another study revealed that in renal tissue (Figure 2), IOPs ameliorated diabetic renal injury in streptozotocin-induced diabetic mice by inhibiting the NF-κB pathway, thereby downregulating the levels of interleukin (IL)-2, IL-2R, and other inflammation-related factors [75].

### 5.3. Immunomodulatory Activity

Immune homeostasis is essential, as dysregulation increases the risk of multiple diseases. With the rising prevalence of suboptimal health, effective immune regulation becomes increasingly important. Studies show that IOPs have bidirectional immunomodulatory effects. They boost host defenses when immunity is low and curbs tissue damage from overactive responses.

Current understanding identifies several key mechanisms for IOP immunomodulation: immune cell activation, enhancement of immune signaling pathways, anti-inflammatory effects, and restoration of immune balance restoration. Experimental evidence indicated that alkaline soluble polysaccharides AIOPA significantly promoted lymphocyte proliferation while also enhancing phagocytic activity and increasing NO, inducible NO synthase (iNOS), and tumor necrosis factor-α (TNF-α) production [76]. In murine RAW264.7 macrophages, polysaccharide PFIO extracted from the fruiting body stimulated the production of NO, reactive oxygen species, and TNF-α. Further mechanistic investigations revealed that this activation was mediated through the mitogen-activated protein kinase (MAPK) and NF-κB signaling pathways, a process potentially dependent on Toll-like receptor 2 recognition [77]. In colitis model mice, IOPs maintained the immature phenotype of bone marrow-derived dendritic cells, which promoted the polarization of CD4^+^ T cells towards anti-inflammatory regulatory T cells (Treg cells) while suppressing the generation of pro-inflammatory Th1 and Th17 cells, thereby further supporting immune balance [78]. Further research demonstrated that IOPs modulated the Th17/Treg equilibrium by downregulating Th17-related transcription factors STAT3 and RORγt while upregulating the Treg-associated factor Foxp3. Concurrently, IOPs interrupted Toll-like receptor 4 (TLR4)/NF-κB signaling to stabilize this balance, as shown in Figure 2 [79].

### 5.4. Antitumor Activity

Cancer represents a major public health threat, driving the exploration of novel antitumor agents. Chinese herbal bioactives, notably from *Inonotus obliquus*, are attracting intense interest for their antitumor activity. *Inonotus obliquus* has a long history of use in traditional medicine. Its polysaccharide-rich extracts exhibit potent antitumor activity in vitro [80].

Extensive studies identified IOPs as major antitumor agents with broad-spectrum activity. In vivo, IOPs dose-dependently inhibited murine Jurkat cell proliferation [81]. In vitro, IOPs suppressed proliferation in diverse human and murine tumor lines, including murine lung cancer cells (LLC1), murine B16-F10 melanoma cells, human osteosarcoma cells (MG-63, U2OS), human adenocarcinoma cells (A549), human colon cancer cells (SW620), and human non-small cell lung carcinoma cells, among others [57,82,83,84,85]. In colonic tissue, as illustrated in the Figure 2, IOPs lowered the IL-6, TNF-α, and COX-2 levels, increased NLRP3 inflammasome expression and IL-1β/IL-18 release, and mitigated pathology to combat colon cancer [83]. In lung tissue (Figure 2), IOPs induced apoptosis in cancer cells both in vitro and in vivo by activating adenosine monophosphate activated protein kinase (AMPK), reducing mitochondrial membrane potential, and evidenced by the downregulation of B-cell lymphoma-2 (Bcl-2), upregulation of Bax, and proteolytic cleavage of Caspase-3 and poly ADP-ribose polymerase (PARP) [57]. Besides, IOPs have been found to induce moderate cell cycle arrest at the G1 phase, thereby inhibiting cancer cell proliferation [86]. Su et al. [82] showed that IOPs suppressed proliferation, migration, and invasion of MG-63 and U2OS osteosarcoma cells, as shown in Figure 2. Furthermore, IOPs also suppressed the activation of the Akt/mTOR and NF-κB signaling pathways, thereby inhibiting the aberrant expression of PFKFB2, p21, Vimentin, Bcl-2, Cyclin D1, c-Myc, and HER2, thereby regulating the proliferation, migration, invasion, and apoptosis of osteosarcoma cells. Collectively, IOPs exert antitumor effects through multiple mechanisms: immune regulation, apoptosis induction, inhibit cell proliferation, and suppression of tumor migration/invasion.

### 5.5. Antioxidant Activity

Lipid peroxidation in the human body is detrimental to health, primarily due to the generation of free radicals [87]. The antioxidant activity of IOPs is mainly manifested through scavenging oxygen free radicals, modulating signaling pathways, thereby mitigating free radical-induced damage and exerting antioxidant effects. The antioxidant assays demonstrated that the scavenging activities of IOPs against DPPH, hydroxyl, and superoxide anion radicals increased in a concentration-dependent manner. The maximum scavenging rates reached 64.1%, 65.7%, and 34.1%, respectively, indicating that the IOPs possessed potent antioxidant activity [21]. However, a critical interpretation of these data are necessary. The polysaccharide backbone itself, lacking potent redox-active moieties like phenolic hydroxyl groups, has limited capacity for direct electron donation. The significant radical scavenging activity measured in such assays is likely attributable to phenolic compounds (e.g., phenolic acids) that are co-extracted and frequently associated with the polysaccharides through covalent or non-covalent bonds. These associated phenolics are the primary agents responsible for direct radical quenching. In contrast, within biological systems, IOPs exert potent antioxidant effects primarily through indirect, regulatory mechanisms. They function as biological response modifiers that activate endogenous cytoprotective signaling pathways. As depicted in Figure 2, in pancreatic β-cells (RINm5F), IOPs protected against H_2_O_2_-induced oxidative damage. This protection was not achieved via the direct scavenging of H_2_O_2_ but potentially through the modulation of the MAPK and NF-κB signaling pathways and the regulation of apoptosis-related proteins, thereby enhancing cell survival [88].

### 5.6. Hypolipidemic Activity

As living standards have improved, dietary patterns have gradually shifted from low-fat to high-fat, leading to an increased incidence of hyperlipidemia. Studies have shown that *Inonotus obliquus* and its active components can effectively reduce blood lipid levels and improve human health. In animal model studies, the acidic polysaccharide fraction IOP-A reduced the liver and spleen indices, limited hepatic fatty degeneration, and lowered serum levels of triglyceride (TG), total cholesterol (TC), low-density lipoprotein (LDL), aspartate aminotransferase, and alanine aminotransferase while also increasing high-density lipoprotein (HDL). The authors suggested that the lipid-lowering effect of IOP-A may be primarily mediated through the promotion of cholesterol metabolism and the regulation of related proteins such as CYP7A1, LXRα, SR-B1, and ABCA1 [89]. In hepatic tissue, as depicted in Figure 2, IOP reduced the body weight and blood lipid levels by activating AMPK and suppressing the expression of lipogenic genes, including sterol regulatory element binding protein (SREBP-1C), fatty acid synthases (FAS), and ACC, in the mouse liver [62].

Furthermore, elevating short-chain fatty acid (SCFA) levels is widely recognized as one of the key therapeutic pathways mediating the effects of multiple gut microbiome-targeted strategies, such as dietary adjustments, prebiotic or probiotic supplementation, and fecal microbiota transplantation (FMT) [90]. Through microbial fermentation, IOPs facilitate the production of SCFAs, which serve as critical mediators of its metabolic benefits. Yang et al. [29] showed that IOPs promoted SCFA-producing bacteria, thereby enhancing the SCFA levels and restoring intestinal microecological balance. In the gut, as illustrated in Figure 2, these SCFAs activated the AMPK pathway, stimulated glucagon-like peptide-1 (GLP-1) secretion, and suppressed hepatic lipogenesis, thereby effectively improving hyperlipidemia [91]. Beyond SCFAs, IOPs influence other microbial metabolites, such as bile acids, which play critical roles in lipid and glucose metabolism. Bile acids, synthesized hepatically by enzymes such as CYP7A1, facilitate fat absorption and metabolic regulation. In the liver, as shown in Figure 2, IOPs exerted hypolipidemic effects through CYP7A1-mediated cholesterol metabolism activation and SREBP-1C inhibition, thereby modulating lipid homeostasis [92]. This suggests that IOPs regulate hyperlipidemia partially through gut microbiota-influenced bile acid pathways.

### 5.7. Antiviral Activity

Viral diseases represent a major public health concern and pose a continuous risk of developing into future pandemics. Natural compounds derived from herbal medicines and medicinal mushrooms offer valuable resources for the development of novel antiviral drugs. IOPs have been reported to possess broad-spectrum antiviral activity against several feline viruses, such as feline influenza virus, feline calicivirus, feline panleukopenia virus, feline herpesvirus type 1, and feline infectious peritonitis virus. The proposed mechanism involves interference with viral particles and/or cellular receptors, thereby inhibiting viral entry into host cells [93]. In the lung (Figure 2), molecular docking analyses further indicated that specific components of *Inonotus obliquus*, including β-glucan, galactomannan, and betulinic acid, can strongly bind to the S1 carboxy-terminal domain (S1-CTD) of the SARS-CoV-2 receptor-binding domain (RBD), particularly at residues TRP-436, ASN-437, and ASN-440, suggesting a potential mechanism for inhibiting SARS-CoV-2 infection [94].

### 5.8. Other Activities

In addition to the aforementioned effects, IOPs also exhibit a range of pharmacological activities, including anti-fatigue, anti-aging, anti-inflammatory and anti-*Toxoplasma gondii* (*T. gondii*) effects. In the context of anti-fatigue activity, in liver, skeletal muscle, and brain tissue (Figure 2), studies have shown that IOP administration significantly extended the swimming endurance of mice, accompanied by increased glycogen reserves in the liver and skeletal muscle, along with reduced levels of blood lactic acid (BLA), serum urea nitrogen (BUN), and lactate dehydrogenase (LDH) [95]. Further investigation suggested that these effects may be mediated through decreased concentrations of 5-hydroxytryptamine (5-HT) in the mouse brain [96]. In inflammatory models, IOPs have been found to significantly downregulate key pro-inflammatory cytokines, including IL-1β, IL-6, and TNF-α while also suppressing NO production in mouse macrophages [97]. Additionally, against *T. gondii* infection in hepatic tissue (Figure 2), IOPs demonstrated potent anti-inflammatory and antioxidant activities. The anti-inflammatory effect was mediated via the TLRs/NF-κB pathway through suppression of the pro-inflammatory cytokines TNF-α, interferon-γ (IFN-γ), IL-6, IL-1β, and IL-4. The antioxidant effects are achieved through a combination of direct and indirect mechanisms. While the observed upregulation of nuclear factor erythroid 2-related factor 2 (Nrf2) and heme oxygenase-1 (HO-1) represents an indirect, cell-signaling mediated pathway that enhances the endogenous antioxidant defense system, the direct free radical scavenging activity (e.g., against DPPH) often reported for IOP extracts is primarily attributed to phenolic compounds (e.g., phenolic acids) that are non-covalently associated or covalently conjugated to the polysaccharide chains. These associated phenolics can directly donate electrons to quench radicals. Furthermore, the polysaccharide components may contribute to the overall antioxidant capacity by chelating pro-oxidant metal ions. Thus, the net antioxidant effect in vivo, as indicated by the reduction in MDA and NO levels and the elevation of GSH and SOD [27], likely results from the synergistic action of polysaccharide-induced cytoprotective signaling and the direct redox activity of co-extracted phenolic constituents.

## 6. Toxicity of IOPs

In evaluating the biosafety of IOPs, multiple studies have consistently demonstrated a favorable toxicological profile. In vitro assessments using the MTT assay demonstrated that the endo-polysaccharide generally lacks direct cytotoxic effects; it did not affect most cancer cell lines or normal cells, with only the Hur7 and MCF-7 lines showing susceptibility, while melanoma cells remained unaffected even at high concentrations (200 μg·mL^−1^) [58]. Complementary in vivo studies corroborate these findings. Acute oral toxicity tests classified water-soluble IOPs as very low toxicity substances, causing no observable lesions in mice at a limit dose of 5000 mg/kg body weight [98]. Furthermore, sub-acute toxicity evaluation of a novel IOP-chromium(III) complex (UIOPC) in normal mice showed no mortality, no adverse effects on organ indices or serum profiles, and no histopathological damage to major organs such as the liver, kidney, and pancreas. Hepatic oxidative stress markers also remained unchanged, confirming the absence of sub-acute toxicity. Collectively, these findings indicate that *Inonotus obliquus* polysaccharides and their derivatives exhibit minimal to no toxicity in both cellular and animal models [99]. However, there remain gaps in the current research on the toxicity of IOPs, particularly regarding genotoxicity, structure–activity relationships, differences among various sources, long-term chronic toxicity, and safety evaluations in special populations. These knowledge gaps hinder their translation into standardized pharmaceuticals or supplements. Therefore, long-term toxicological studies are still required.

## 7. Application

As above-mentioned, IOPs, derived from a rare natural medicinal and edible mushroom, exhibit a variety of biological activities such as intestinal flora regulation, hypoglycemic, immunomodulatory, antitumor, antioxidant, hypolipidemic, and antiviral effects, demonstrating significant application value in the fields of medicine and food science. Consequently, a growing number of functional foods based on IOPs have been developed. As reported by Xue [19], yogurt fermented with IOPs demonstrated enhanced digestibility and absorption, along with superior nutritional value and health benefits compared to conventional yogurt. Furthermore, livestock studies showed that *Inonotus obliquus* was rich in immune-supportive β-glucan, which may improve poultry growth [100].

Owing to its diverse biological activities, IOPs hold strong potential for multi-target health products. In metabolic health, their hypoglycemic and lipid-lowering effects suit functional foods and medical nutrition for diabetes or obesity. For immune support, IOPs can be added to supplements or beverages to boost immunity. Their strong antioxidant profile also positions them as candidates for anti-aging nutraceuticals and cosmeceuticals. Additionally, IOPs’ gut-microbiota modulation supports their use as a prebiotic in synbiotics for digestive health.

## 8. Conclusions

IOPs are a structurally diverse and pharmacologically promising class of natural macromolecules. This review provides a comprehensive synthesis of the current knowledge spanning their preparation, structural characteristics, structure–activity relationships, biological activities, toxicity, and potential applications. Significant progress has been made in developing extraction and purification methodologies, yet achieving standardized, high-purity fractions suitable for industrial-scale production remains a key challenge. The elucidation of precise chemical structures is inherently difficult due to the structural diversity and heterogeneity of IOPs, representing a fundamental analytical frontier in the field.

A central theme of this review is the critical link between structure and function. Evidence confirms that specific structural attributes—including molecular weight, glycosidic linkage patterns (e.g., (1 → 3)/(1 → 6)-β-Glc), monosaccharide composition, and chemical modifications—collectively determine their biological effects. Furthermore, the prebiotic function of IOPs—achieved by modulating gut microbial ecology, stimulating the production of metabolites such as SCFAs, strengthening the intestinal barrier, and potentially exhibiting sex-specific effects—constitutes a core mechanism mediating many of their systemic benefits. IOPs exhibit a broad spectrum of biological effects, including gut microbiota modulation, hypoglycemic and hypolipidemic activities, immunomodulation, antitumor potential, antioxidant capacity, and antiviral activity. Importantly, toxicological assessments to date suggest a favorable safety profile, supporting their exploration in functional foods and nutraceuticals. The translation of this potential, however, hinges on overcoming the current limitations in structural characterization, mechanistic understanding, and clinical validation. In summary, while IOPs hold considerable promise, realizing their full application requires an integrated approach that connects advanced analytics, unambiguous structural elucidation, and targeted biological studies. The insights synthesized herein, particularly on structure–activity relationship and gut microbiota crosstalk, provide a foundational roadmap for future research aimed at developing IOP-based products for health promotion and disease management.

## 9. Perspectives

Despite considerable progress, several challenges must be addressed to fully realize the potential of IOPs in therapeutics and nutraceuticals. Future research should prioritize the following directions:

1. Scalable and sustainable production processes must be developed to enable the industrial application of IOPs. Current extraction and purification methods remain largely confined to laboratory scales, hindering the production of high-purity IOPs. Future work must focus on optimizing these processes, integrating green technologies to improve yield, reduce cost, and enable the scalable production of standardized preparations.
2. Fine structural characterization of homogeneous IOPs is fundamental to understanding their bioactivity. The precise structural basis governing their key bioactivities is still poorly defined. Employing advanced analytical techniques to isolate such fractions and determine their exact primary and three-dimensional structures is a crucial prerequisite for rational drug design and clinical translation.
3. The molecular mechanisms underlying IOP–gut microbiome interactions require in-depth clarification. Research on this crosstalk is still in its infancy. Key unanswered questions include the specific microbial taxa and metabolic pathways involved, and the subsequent signaling to the host. Integrated multi-omics approaches are essential to decipher these complex mechanisms.
4. A robust translational pipeline from preclinical studies to human trials is imperative for clinical development. Most current evidence derives from in vitro and rodent studies. Future work must validate safety and efficacy in relevant large animal models, followed by well-designed clinical trials to evaluate therapeutic efficacy, optimal dosage, and long-term safety in target human populations.

In short, concerted efforts to advance the scalable production, fine structural elucidation, mechanistic understanding, and clinical translation of IOPs will be crucial to fully realize their potential as functional foods and pharmaceuticals.

## Figures and Tables

**Table 2:** Common separation and purification methods for polysaccharides and their characteristics.

Purpose	Method	Principle	Advantages	Disadvantages
Deproteinization	Sevage method	Protein denaturation and precipitation at the interface of an organic (chloroform/n-butanol) and aqueous phase	Mild reaction conditions; effective for deproteinization	Time-consuming; substantial polysaccharide loss; high solvent consumption and risk of residual solvent; less effective for tightly bound proteins
	TCA method	Protein conformation alteration and irreversible precipitation	Simple operation procedure; low solvent consumption	Risk of structural alteration
	Enzymatic methods	Proteolytic enzymes degrade proteins	Mild reaction conditions; high polysaccharide retention rate	High enzyme cost; potential for enzyme inactivation and residual enzyme in the product; may impair glycoprotein complexes
Depigmentation	Activated carbon adsorption	Physical adsorption and chemical adsorption	Simple operation; cost-effective; eco-friendly	Slow kinetics; significant polysaccharide loss; difficult to remove carbon particles completely
	Hydrogen peroxide oxidation method	Oxidation by H_2_O_2_	High decolorization efficiency	Potential for oxidation and degradation of the polysaccharide backbone
	Resin adsorption	Physical adsorption and chemical adsorption	High capacity and selectivity; rapid kinetics; reusable	Less effective for highly viscous solutions or for pigments with similar polarity to the target polysaccharides
Fractionation	Ion-exchange chromatography	Depending on the charge	High resolution for charged polysaccharides; high purity of fractions	Requirement for buffer exchange; high cost; risk of column or polysaccharide damage from extreme pH
	Gel chromatography	Depending on molecular size	High resolution by Mw; mild, non-denaturing conditions	High cost; low loading capacity; significant challenges for process scale-up

## Data Availability

No new data were created or analyzed in this study. Data sharing is not applicable to this article.
